# Loading dendritic cells from HIV-1 infected patients with PLA-p24 nanoparticles or MVA expressing HIV genes induces HIV-1-specific T cell responses

**DOI:** 10.1186/1742-4690-9-S2-P247

**Published:** 2012-09-13

**Authors:** N Climent, S Munier, N Piqué, F García, V Pavot, A León, C Primard, L Miralles, C Rovira, V Casanova, P McCormick, JM Gatell, B Verrier, T Gallart

**Affiliations:** 1 Hospital Clinic, Barcelona, Spain; 2 Institut de Biology et Chimie des Protéines, FRE3310 CNRS/UCBL, Lyon, France; 3 Dept. Microbiology and Parasitology, Pharmacy Faculty, U of Barcelona, Barcelona, Spain; 4 Department of Biochemistry and Molecular Biology, Faculty of Biology, Barcelona, Spain; 5 Dept. Biochemistry and Molecular Biology, Faculty of Biology, Barcelona, Spain

## Background

The evaluation of the interaction of new immunogens with dendritic cells is important for new vaccine strategies. We used polylactic acid (PLA) colloidal biodegradable particles, coated with HIV Gag antigens (p24), and Modified Vaccinia Ankara (MVA) expressing Gag or Tat, Nef and Rev in an ex vivo model of human monocyte-derived dendritic cells (MDDC) to compare two different loading strategies,either viral or synthetic derived.

## Methods

We have assessed the interaction of PLA nanoparticles bearing p24 (PLA-p24) or MVA expressing Gag or Tat, Nef and Rev with MDDC of HIV-1 infected patients and the capacity of these cells to process and present the HIV-1 antigens to autologous human T cells from HIV-1-infected patients.

## Results

PLA-nanoparticles were captured by 98% of MDDC from HIV-1 infected patients, without deleterious effects for the cells. Capture of PLA-p24 induced a slight degree of MDDC maturation, cytokine and chemokine secretion and migration towards a gradient of CCL19 chemokine. After complete maturation induction of PLA-p24-pulsed MDDC, maximal expression of maturation markers was observed, together with a strong migration towards a gradient of CCL19 chemokine. PLA-p24-loaded MDDC were able to induce HIV-specific T cell proliferation (two-fold higher for CD4+ than CD8+) and cytokine secretion (IFN-γ and IL-2). Upon exposure to MVA-gag, MDDC produced cytokines and chemokines and maintained their capacity to migrate to a gradient of CCL19. MDDC infected with MVA-gag, MVA-gag trans-membrane and MVA-nef were able to induce HIV-specific CD8+ proliferation and secretion of IFN-γ, IL-2, IL-6 and TNF-α.

## Conclusion

These findings support the use of both HIV antigens (PLA particles carrying HIV antigens or MVA expressing HIV genes) as anti-HIV vaccines.

